# Recursive Cluster Elimination based Rank Function (SVM-RCE-R) implemented in KNIME

**DOI:** 10.12688/f1000research.26880.2

**Published:** 2021-01-05

**Authors:** Malik Yousef, Burcu Bakir-Gungor, Amhar Jabeer, Gokhan Goy, Rehman Qureshi, Louise C. Showe

**Affiliations:** 1 Zefat Academic College, Zefat, Israel; 2 Department of Computer Engineering, Faculty of Engineering, Abdullah Gul University, Kayseri, Turkey; 3 The Wistar Institute, Philadelphia, PA, USA

**Keywords:** clustering, machine learning, recursive, gene expression, ranking, grouping, KNIME

## Abstract

In our earlier study, we proposed a novel feature selection approach, Recursive Cluster Elimination with Support Vector Machines (SVM-RCE) and implemented this approach in Matlab. Interest in this approach has grown over time and several researchers have incorporated SVM-RCE into their studies, resulting in a substantial number of scientific publications. This increased interest encouraged us to reconsider how feature selection, particularly in biological datasets, can benefit from considering the relationships of those genes in the selection process, this led to our development of SVM-RCE-R.  SVM-RCE-R, further enhances the capabilities of  SVM-RCE by the addition of  a novel user specified ranking function. This ranking function enables the user to  stipulate the weights of the accuracy, sensitivity, specificity, f-measure, area  under the curve and the precision in the ranking function This flexibility allows the user to select for greater sensitivity or greater specificity as needed for a specific project.

The usefulness of SVM-RCE-R is further supported by development of the maTE tool which uses a similar approach to identify microRNA (miRNA) targets. We have also now implemented the SVM-RCE-R algorithm in Knime in order to make it easier to applyThe use of SVM-RCE-R in Knime is simple and intuitive and allows researchers to immediately begin their analysis without having to consult an information technology specialist. The input for the Knime implemented tool is an EXCEL file (or text or CSV) with a simple structure and the output is also an EXCEL file. The Knime version also incorporates new features not available in SVM-RCE.

The results show that the inclusion of the ranking function has a significant impact on the performance of SVM-RCE-R. Some of the clusters that achieve high scores for a specified ranking can also have high scores in other metrics.

## Introduction

The application of a variety of new technologies for measuring gene expression has generated publicly available datasets with very high feature dimensionalities (tens of thousands of genes) ^1, 2^. Because expression of certain groups of genes can be functionally related, they can be grouped according to a specific metric, which can be defined by the biological processes and interactions the group represents. Since most of the existing feature selection approaches have been borrowed from the field of computer science and statistics, they fail to consider the associations between gene expression features. We now propose to address that issue. In our initial study we suggested an algorithm called SVM-RCE ^3^, where genes were grouped using a k-means based clustering algorithm. Our following study, SVM-RNE ^4 ^ incorporated the possibility of grouping subsets of genes according to gene sub-networks. Our recent tool maTE ^5^ suggested an alternative grouping based on microRNA targets and replaced k-means clustering with ensemble clustering ^6^.

Sahu and Mishra ^7^ have stressed the weakness of Signal-to-Noise Ratio (SNR) and t-statistics, which are widely used for gene rankings in the analysis of gene expression data, as using SNR and t-statistics as filtering techniques will likely select redundant features. They instead suggest that the genes are first grouped into clusters based on the similarities of their expression values, followed by the application of different filtering techniques to rank the genes in each cluster. The assigned ranks were then used to select the most informative genes from each cluster resulting in improved classification. The problem of dealing with clusters of features or groups of correlated features, in remote sensing data sets, was also recently addressed by Harris and Niekerk ^8^. They stress the importance of first clustering the features by affinity propagation, and then applying a ranking function to overcome the weakness of the traditional feature selection approaches, which are likely to result in the selection of sub-optimal features. Therefore, the ranking function we propose is founded upon a process of assigning weights to the various clusters based on their performance metrics. This allows the user to specify which metric they want to focus on depending on their needs. The implementation of the ranking function is explained in further detail in the ranking function section.

## Methods

### The SVM-RCE workflow

The SVM-RCE algorithm can be described by three main steps:

1. The Clustering step combines the genes, based on expression, into groups using a clustering algorithm such as K-means. The merit of this step is to put genes with similar expression patterns into one cluster in order to deal with them together. In general, we refer to this step as a grouping function.
2. The Rank step ranks each cluster using a function we have used in the SVM-RCE ^3^ using Rank(X(S), f, r) as the average accuracy of the linear SVM over the data X represented by the S genes computed as f-folds cross validation repeated r times. We set *f* to 3 and *r* to 5 as default values (See Pseudocode 1).
3. The RCE step removes the lower ranked clusters of genes and can be implemented to remove one cluster or a percentage of clusters as specified by the researcher, e.g. removing the lower 10% of the clusters.

We have applied the step of recursive cluster elimination based on the hypothesis that the clustering algorithm will generate new sets of clusters and that some of the genes will move between clusters and we have shown this to be the case.

Pseudocode 1. **The Ranking method R(), a main component of the SVM-RCE**

**Ranking Algorithm - *R(X _s_,M,f,r)***

   
**X**_s_: any subset of the input gene expression data X, the features are gene expression values

   
**M { *m _1_,m _2_,...,m _p_*}** is a list of groups produced by k-means.

   
***f*** is a scalar (0≤ *f*≤1): split into train and test data

   
**r:** repeated times (iteration)

   
**res={}** for aggregating the scores for each *m _i_*

**Generate Rank for each** ***m _i_, Rank(m _i_):***

  For each *m _i_* in M

  
*sm _i_*=0;

Perform *r* times (here r=5) steps 1–5:
1. Perform stratified random sampling to split X _s_ into train X _t_ and test X _v_ data sets according to *f* (here 80:20)
2. Remove all genes (features) from X _t_ and X _v_ which are not in the group *m _i_*
3. Train classifier on X _t_ using SVM
4. *t* = Test classifier on X _v_ –calculate performance of test data
5. *sm _i _= sm _i_ + t;*

  
*Score(m _i_)*= *sm _i_* / *r* ; Aggregate performance

  
*res*= $\cup_{i=1}^{p}score\left(m_{i}\right)$

**Output**

*Return res ( res = {Rank(m _1_),Rank(m _2_),…,Rank(m _p_)} )*

### Incorporation of novel ranking function

The algorithm of Recursive Cluster Elimination ^3^ considers clusters of similar features/genes and applies a rank function to each group as described in Pseudocode 1. Since we are using the clustering algorithm k-means we refer to these groups as clusters, but it could be any other biological or more general function that groups the particular features, such as KEGG pathways or microRNA targets, as we have suggested in several other studies ^4, 5^. As illustrated in Pseudocode 1, in the original code of SVM-RCE we used the accuracy which was the performance as the determinant for ranking the clusters. The data for establishing that ranking was divided between training and testing. The data represented by each gene/feature is then assigned to a specific cluster and the rank function is then applied as the mean of *r* repeat times of the repeated training and the testing performance while recording different measurements of accuracy (sensitivity, specificity, etc.).

In this new version implemented in Knime ^9^ we have incorporated more user specific ranking function. The user provides the weights of the following ranking function that correspond to the mean of each measurement achieved by the *r* times of the internal:

$$R\left(w_{1},w_{2},w_{3},w_{4},w_{5},w_{6}\right)=w_{1}\times acc+w_{2}\times sen+w_{3}\times spe+w_{4}\times fm+w_{5}\times auc+w_{6}\times prec$$

Where the *acc* is the accuracy, *sen* is the sensitivity, *spe* is the specificity, *fm* is the f-measurement, *auc* is the area under the curve and *prec* is precision.

The coefficient weights represent the importance of each measurement for searching those clusters of genes that contribute to the final performance requirements. For example, if the user is interested in achieving greater specificity than sensitivity, the user would choose weights of 0.7 for the parameter *spe* and 0.3 for *sen*, stating that he is searching for clusters of genes that contribute to high specificity. However, one can also choose all the weights to be zero, with the weight of accuracy is set as 1, the rank function will then only rely on the accuracy.

### Implementation in Knime

**Figure 1.**
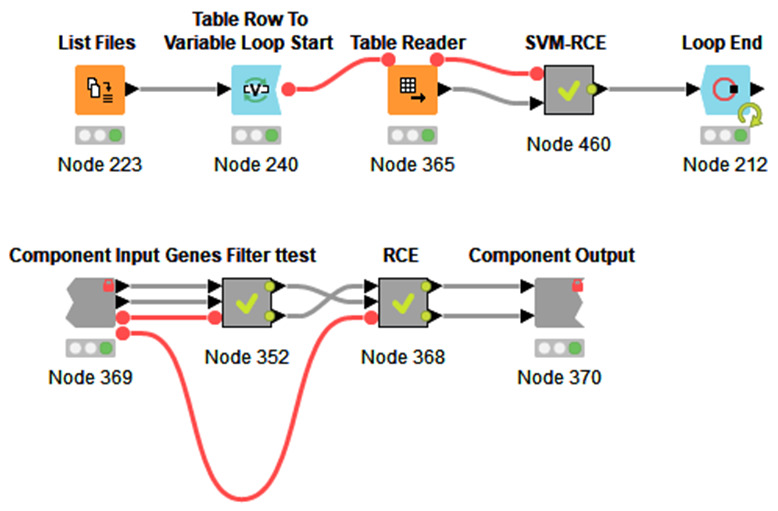
( **a**) The main Knime workflow for SVM-RCE-R that can be executed on multiple input files. ( **b**) The internal meta-node SVM-RCE-R that consists of two components.

We have used the free and open-source platform Knime ^10^ for re-coding SVM-RCE ( Figure 1– Figure 3) due to its simplicity and useful graphical presentations. Knime is a highly integrative tool that allows the user to include other programming languages such as R, Python and Java. In addition, one can also add external packages as such WEKA, H2O and so on. Figure 1 presents the workflow that includes SVM-RCE-R as a meta-node. The workflow can be executed on multiple input files. The node “List Files” will be indicated on the folder that has the input files. The workflow loops through those files and runs the SVM-RCE-R meta-node. The “Loop End” is also collecting specific results that can be subjected to further analysis.

**Figure 2.**
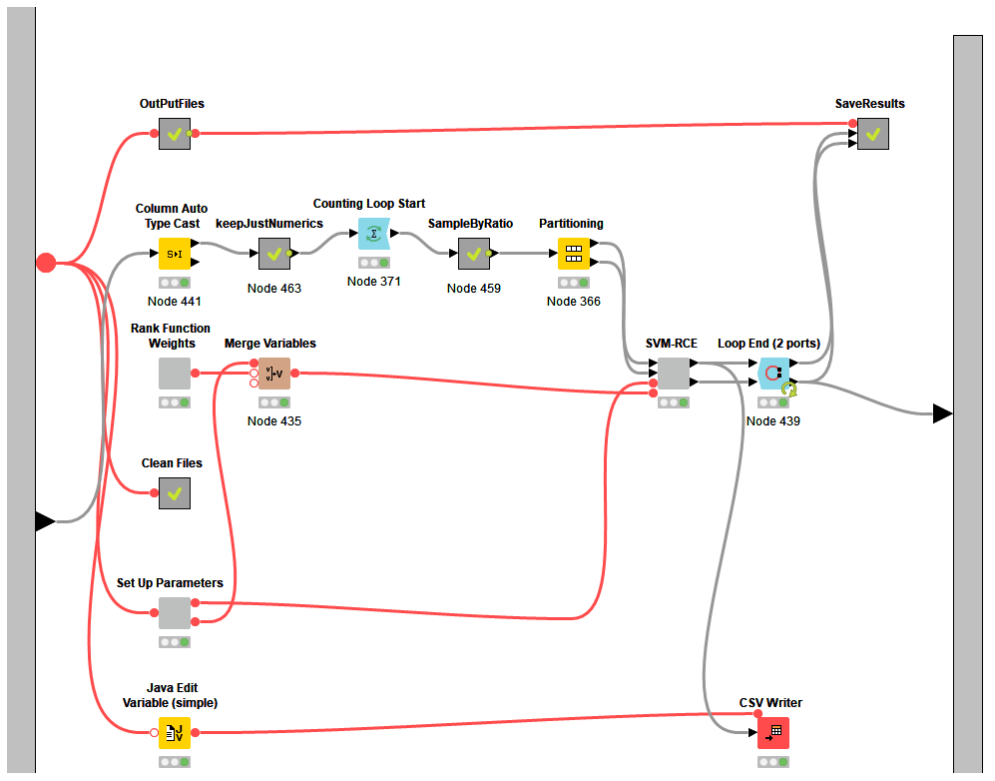
The interface of the SVM-RCE-R.

**Figure 3.**
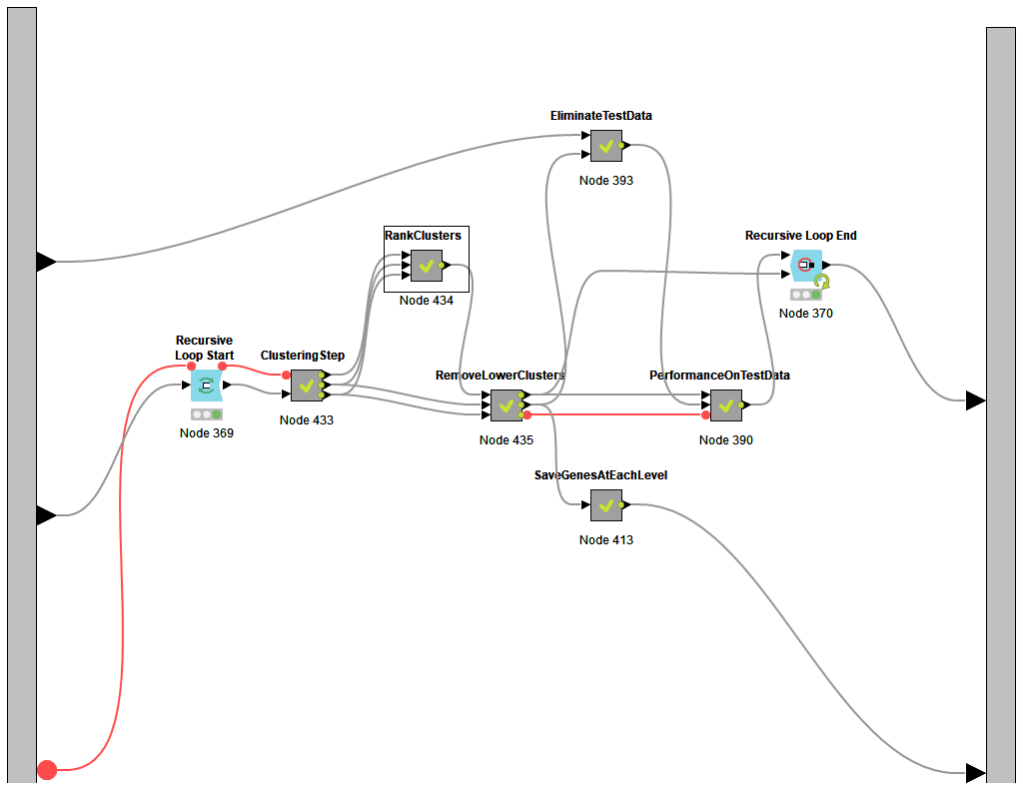
The nodes present in the meta-node SVM-RCE-R.

The SVM-RCE-R meta-node consists of two components (two meta-nodes). The meta-node “Genes Filter t-test” ( Figure 1b) is used to reduce the dimension of the features by applying the t-test to the training part of the data. Following that is the RCE component.

The interface of the SVM-RCE-R is presented in Figure 2. This part of the tool is used to set different parameters. The user can specify the number of iterations for Monte Carlo cross-validation (MCCV) by configuring the node “Counting Loop Start”. MCCV is the process of randomly selecting (without replacement) some fraction of the data to form the training set, and then assigning the rest to the test set. The node “Partitioning” is used to specify the ratio of the training/testing splitting.

The most important component “Rank Function Weights” is related to the rank function R(), where the user specifies the values of the weights w1, w2, .., w6. We show in the results section that these values have an impact on the performance of the SVM-RCE-R.

Figure 3, meanwhile, shows nodes present in the meta-node SVM-RCE. It is designed so that it follows the pseudocode, thereby making it user-friendly.

### Operation

The workflow was developed in KNIME which is compatible with Mac, Linux and Windows OS. We would recommend using a quad core CPU with at least 8 GB of RAM to run the workflow. Moreover, users will need to install Python 3 and R environments, Anaconda is recommended for the installation of Python 3 meanwhile R > 1.5 should be installed with Rserve package which can be found at https://cran.r-project.org/web/packages/Rserve/index.html. 

### Gene expression data

12 human gene expression datasets were downloaded from the Gene Expression Omnibus at NCBI ^11^. For all datasets, disease (positive) and control (negative) data were available ( Table 1). All of the datasets are gene expression data with different number of samples and were used as is in our workflow. The columns of the datasets indicate the sample identification code and the rows contain the names of the genes. Moreover, the sample input datasets can be found in the underlying data repository. Those 12 datasets served to test the SVM-RCE-R tool and to compare its performance with two other approaches; the filter and embedded approaches ^12, 13^. The first approach performs feature selection using information gain (SVM-IG) on the training part while the second approach is compared with SVM with recursive feature elimination (SVM-RFE) ^14^. We have also implemented a workflow for SVM-RFE that is based on the Scikit-learn package ^15^ in Knime. 

**Table 1.** Description of the 12 data sets used in our study. The data sets are obtained from GEO. Each entry has the GEO code the name of the data, the number of samples and the classes of the data.

GEO Accession	Title	Sample count	Classes
GDS1962	Glioma-derived stem cell factor effect on angiogenesis in the brain	180 pos=157 neg=23	non-tumor=23 (neg) astrocytomas=26 (pos) glioblastomas=131 (pos)
GDS2519	Early-stage Parkinson's disease: whole blood	105 pos=50 neg=55	healthy control=22 (neg) neurodegenerative disease control=33 (neg) Parkinson disease=50 (pos)
GDS3268	Colon epithelial biopsies of ulcerative colitis patients	202 pos=73 neg=129	normal=73 ulcerative colitis=129
GDS2547	Metastatic prostate cancer (HG-U95C)	164 pos=75 neg=89	normal=75 tumor=89
GDS5499	Pulmonary hypertensions: PBMCs	140 pos=99 neg=41	control=41 (neg) idiopathic pulmonary arterial hypertension=30 (pos) scleroderma-associated pulm. arterial hypert=42 (pos) systemic sclerosis (SSc) without pulm. hypert=19 (pos) SSc, interstitial lung disease & pulm. hypert=8 (pos)
GDS3646	Celiac disease: primary leukocytes	132 pos=110 neg=22	healthy control=22 celiac disease=110
GDS3874	Diabetic children: peripheral blood mononuclear cells (U133A)	117 pos=93 neg=24	healthy=24 type 1,2 diabetes=93
GDS3837	Non-small cell lung carcinoma in female nonsmokers	120 pos=60 neg=60	lung cancer=60 control=60
GDS5037	Severe asthma: bronchial epithelial cell	108 pos=88 neg=20	mild asthma=50 control=20 severe asthma=38
GDS4516_ 4718	Colorectal cancer: laser microdissected tumor tissues Colorectal cancer: homogenized tumor tissues	148 pos=104 neg=44	laser microdissected tumor tissues=104 homogenized tumor tissues=44
GDS3900	Fear conditioning effect on the hybrid mouse diversity panel: hippocampus and striatum	198 pos=100 neg=98	hippocampus=100 (pos) striatum=98 (neg)
GDS3929	Tobacco smoke effect on maternal and fetal cells	183 pos=128 neg=55	non-smoker=128 (pos) smoker=55 (neg)

## Results

We have tested SVM-RCE-R on the aforementioned datasets and used the performance results to verify our new ranking function. For the comparison of the three approaches, we have considered five datasets (GDS1962, GDS3646, GDS3874, GDS3900, GDS5499) as listed in Table 2. We have applied SVM-RCE-R, obtaining the performance over 100 iterations. At each iteration we have split the data into 90% for training and 10% for testing. The average of all different performance measurements is then aggregated. For additional comparison we refer to the first study published about SVM-RCE-R ^3^.

**Table 2.** Comparison results between SVM-RCE-R, SVM-RFE and SVM-IG including the standard deviation.

	Information		SVM-RCE-R				SVM-RFE				SVM-IG			
	#samples	#genes	Sensitivity	Specificity	Accuracy	#Genes	Sensitivity	Specificity	Accuracy	#Genes	Sensitivity	Specificity	Accuracy	#Genes
**GDS1962**	180	54613	0.94 ± 0.09	1.00 ± 0.00	0.96 ± 0.07	97.00 ± 39.11	0.93 ± 0.13	0.97 ± 0.06	0.94 ± 0.08	100	0.90 ± 0.12	0.97 ± 0.11	0.93 ± 0.08	100
**GDS3646**	132	22185	0.80 ± 0.19	0.50 ± 0.33	0.71 ± 0.19	108.70 ± 45.91	0.74 ± 0.32	0.64 ± 0.21	0.67 ± 0.17	100	0.63 ± 0.24	0.67 ± 0.25	0.65 ± 0.18	100
**GDS3874**	117	22283	0.82 ± 0.18	0.53 ± 0.17	0.71 ± 0.13	112.80 ± 38.37	0.84 ± 0.16	0.65 ± 0.24	0.70 ± 0.16	100	0.79 ± 0.46	0.79 ± 0.10	0.79 ± 0.24	100
**GDS3900**	198	25697	1.00 ± 0.00	1.00 ± 0.00	1.00 ± 0.00	141.20 ± 67.81	1.00 ± 0.00	1.00 ± 0.00	1.00 ± 0.00	100	0.99 ± 0.02	1.00 ± 0.01	0.99 ± 0.01	100
**GDS5499**	140	49577	0.93 ± 0.08	0.90 ± 0.13	0.92 ± 0.07	145.00 ± 44.54	0.93 ± 0.07	0.90 ± 0.10	0.88 ± 0.08	100	0.90 ± 0.11	0.83 ± 0.14	0.87 ± 0.08	100

The results indicate that SVM-RCE-R outperforms or is equivalent to the other approaches in all the datasets except in determining the specificity for GDS3646 with a case to control ratio of 5 to 1 ^16^ and GDS3874. 

**Figure 4.**
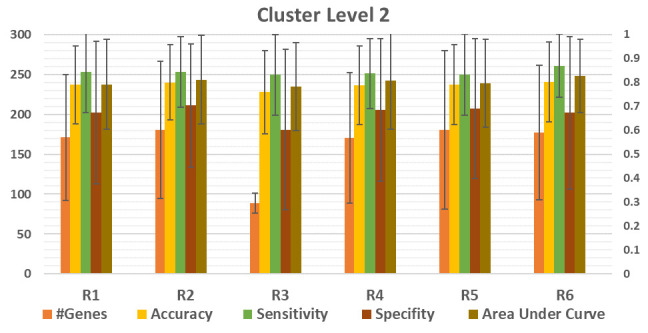
Comparison between the performance of six (R1,..,R6) different functions, listed in Table 3. The average of 100 iterations if computed for different performance measurements for each R1,…,R6 over the 12 datasets. The results of the level of cluster 2 is presented. #Genes is the average number of genes in level 2. The average accuracy, sensitivity, specificity and area under the curve (AUC) is presented for R1,..R6.

We have also considered different values of the rank function *R(w1,w2,w3,w4,w5,w6)* by specifying different values of the measurements weights, w1,..,w6 and have generated six rank functions as listed in Table 3. For each rank function we have applied the SVM-RCE-R obtaining the performance over 100 iterations. At each iteration we have split the data into 90% for training and 10% for testing. The average of all different performance measurements is then aggregated. All of the datasets that are used for the comparison between the performance of six different functions are listed in Table 3 and the results are shown in Figure 4. 

**Table 3.** Different values of weights generating different rank functions. w1,weight of accuracy; w2, weight of sensitivity; w3, weight of specificity; w4, weight of f-measurement; w5, weight of area under the curve.

R1	w1=0.2, w3=0.3, w2= 0.4, w5=0.1
R2	w1=1.0, the rest are zero
R3	w5= 1.0, the rest are zero
R4	w4=1.0, the rest are zero
R5	w3=0.2, w2=0.8
R6	w3=0.8, w2=0.2

Figure 4 shows that there is deviation of the performance measurements for each R. However, we observed that the deviation is clear if we consider each data set individually which will be discussed in further detail in the next section.

### SVM-RCE vs SVM-RCE-R

**Figure 5.**
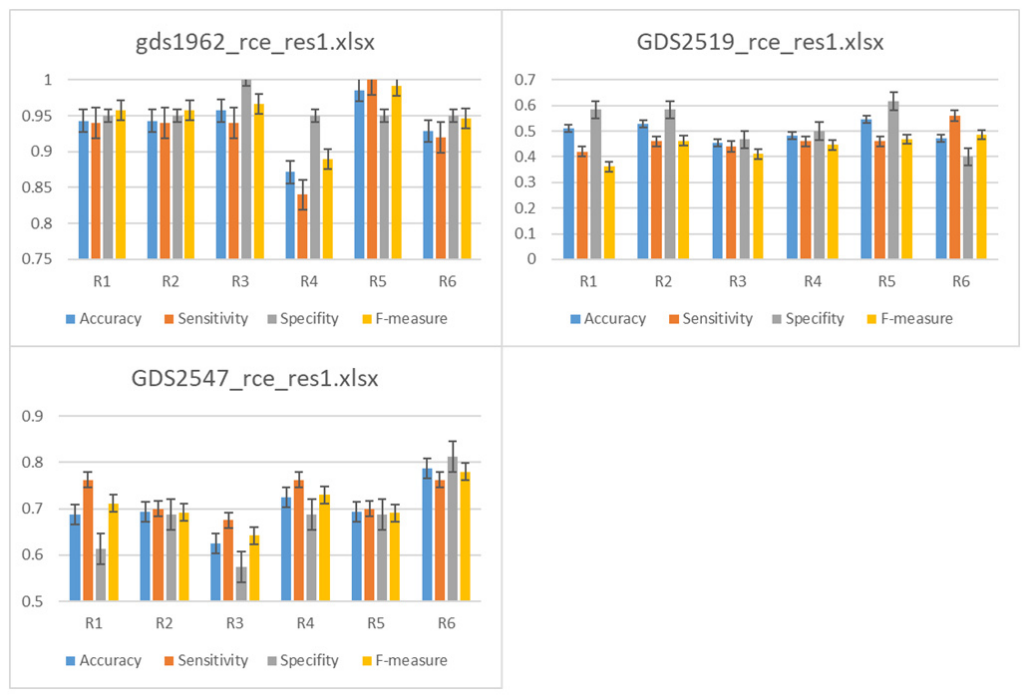
The performance of SVM-RCE-R over different data considering different Ranks functions(R1..R6) where R2 is SVM-RCE. *The results show the increase/decrease of those rank functions on the accuracy, sensitivity and specificity*.

In order to examine the effect of the Rank function, we plotted the results obtained on the cluster level 2 as appears in Figure 5 (See *Underlying data* for all the results for the 12 datasets ^16^) for each data set. 

For example, the accuracy obtained with R5 is significantly greater than R4 by about 12%, while reaching 4%–6% more than the other ranks. Interestingly we are getting a 4% improvement over the standard rank we have been using with the old version of SVM-RCE, which was R2.

GDS2547 data reached an accuracy of ~79% applying R6 and 63% with R3, a difference of 16%, which is about 9% over the standard rank using the previous version SVM-RCE. However, for GDS5037 the max performance obtained with the standard rank R2 reached a difference of 16% over the minimum values reached by R5.

We have calculated the overall difference between the max value of each rank and the R2 that was used in the old version to get 5%.

This indicates that one can dramatically improve the performance of SVM-RCE-R by searching for the optimal values of the weights of the rank function.

**Figure 6.**
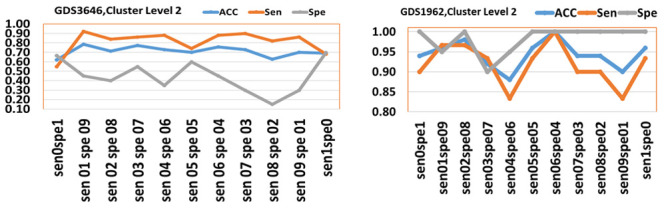
The performance of values of weights for Sen and Spe over (0,0) gradually increased by 0.1 to (1,1). The axes labels are the values, for example sen01spe09 is associated for weight of 0.1 of sensitivity and 0.9 for specificity. The accuracy (ACC), sensitivity (Sen) and specificity (Spe) are plotted.

We also conducted an additional experiment in order to examine the effect of gradually changing the values of sensitivity and specificity weights in the rank function. We ran two experiments on GDS3646 and GDS1962 data considering the values of (1,0) (0,1) (first argument is sensitivity weight while second one is specificity weight) increasing by 0.1 to reach (0,1) for the weights of sensitivity and specificity, respectively. The results are represented in Figure 6 for cluster level 2.

Figure 6 shows that the two graphs are behaving differently over the set of weights, showing that the results depend on the specific data. Interestingly we see that for GDS1962 data, the optimal performance for all measurements is with weight 0.6 and 0.4 for sensitivity and specificity, respectively. Although the maximum accuracy is achieved over (0.1,0.9) weights pair, for GDS3646 data, the specificity at this point is very low and not usable for prediction, while (0.5,0.5) seems to provide reasonable performance for both sensitivity and specificity. Additionally, we have computed the number of common genes by considering the top 50 significant genes for each pair (sen01sep09 vs sen02spe08, …) having on average 11 genes. That is another indication that the rank function also has a significant impact on the list of the significant genes.

## Discussion

As gene expression data sets become more complex, new computational tools that deal with features in a non-traditional way are needed to address this complexity. Our approach does not simply tackle the problem of inherent redundant or correlated features, it also suggests that defining the grouping metrics is equally important when searching that specific feature space that each researcher would like to focus on. Different biological systems/problems can require an output with a greater emphasis on either specificity, sensitivity or overall accuracy. Although specifying a certain metric, for instance, specificity, has higher priority during clustering, there can be cases where the clusters have high values for other metrics, which can be inferred from our results. Therefore, finding the optimal ranking will be one of the topics that we will further focus on. We now provide the capability to decide whether the specific problem being addressed will benefit more from reducing false positives or false negatives.

This new version of RCE now provides the user with the ability to control the analyses and to also design the ranking function that will allow exploration of the data in a way that addresses the specific goals of the analysis. Additionally, since it is easy to change the learning algorithm from SVM or to combine SVM with other machine learning algorithms, it further expands the utility of RCE-R. These additional components will be added to the next version of RCE as well as additional features for optimization procedures. Currently, our program estimates each cluster separately; a future version will combine different numbers of clusters using a search algorithm in order to identify the optimal combination that will return the highest accuracy.

## Data availability

### Source data

Human gene expression datasets from Gene Expression Omnibus, Accession numbers: GDS1962, GDS2519, GDS3268, GDS2547, GDS5499, GDS3646, GDS3874, GDS3837, GDS5037, GDS4516_ GDS4718, GDS3900, GDS3929

### Underlying data

Zenodo: Ajabeer/SVM-RCE-R-results-Omnibus-dataset: Supplementary Data for SVM-RCE-R. https://doi.org/10.5281/zenodo.4327346 ^16^.

This project contains the following underlying data:

- all_res1_clusters.xlsx files (contains the summary of all res_1 files for all 12 datasets for R1-R6)
- logResults.csv files (contains the scoring values and class labels for each run of the SVM-RCE loop for each of the 12 datasets, R1-R6)
- rankedGenes.xlsx files (contains the names of the genes that ranked according to the rank function with their levels, rank function values and scores for each of the 12 datasets, R1-R6)
- res1.xlsx files (contains the mean values of genes and the scoring metrics values calculated: Accuracy, Sensitivity, Specificity, F-measure, AUC, Cohens Kappa, for each cluster level for each of the 12 datasets, R1-R6)
- res2.xlsx files (contains the number of genes for each level, scoring metrics values calculated: Accuracy, Sensitivity, Specificity, F-measure, AUC, Cohens Kappa, for each cluster for each iteration for each of the 12 datasets, R1-R6)
- table files (contains the datasets from GEO expression datasets used as the input file for the workflow)

Data are available under the terms of the Creative Commons Zero "No rights reserved" data waiver (CC0 1.0 Public domain dedication).

## Software availability

The SVM-RCE-R Knime workflow, step-by-step tutorial and a detailed documentation are available on the following web site: https://malikyousef.com/svm-rce-in-knime/

Source code available from: https://github.com/malikyousef/SVM-RCE-R-KNIME

Archived source code at time of publication: https://zenodo.org/record/4066639#.X3sQVlLis2w ^9^

License: GNU General Public License v3.0

Detailed terms and conditions of KNIME can be found at https://www.knime.com/downloads/full-license.
