# Supplementary material for: Recursive Cluster Elimination based Rank Function (SVM-RCE-R) implemented in KNIME
Source: F1000Res. 2021 Jan 5;9:1255. Originally published 2020 Oct 19. [Version 2] doi: 10.12688/f1000research.26880.2 (PMC7802119; doi:10.12688/f1000research.26880.2)
Supplement: Peer review history

# Peer review: Reviewer response for version 2

**DOI:** 10.5256/f1000research.31433.r76608

**Published:** 2021-01-22

**Authors:** Marco Chierici

**Affiliations:** 1 Fondazione Bruno Kessler, Trento, Italy

I am satisfied with the revised version.

Are the conclusions about the tool and its performance adequately supported by the findings presented in the article?

Yes

Is the rationale for developing the new software tool clearly explained?

Yes

Is the description of the software tool technically sound?

Yes

Are sufficient details of the code, methods and analysis (if applicable) provided to allow replication of the software development and its use by others?

Yes

Is sufficient information provided to allow interpretation of the expected output datasets and any results generated using the tool?

Partly

Reviewer Expertise:

Bioinformatics, artificial intelligence, computational biology

I confirm that I have read this submission and believe that I have an appropriate level of expertise to confirm that it is of an acceptable scientific standard.

# Peer review: Reviewer response for version 2

**DOI:** 10.5256/f1000research.31433.r76606

**Published:** 2021-01-18

**Authors:** Abhishek Kumar

**Affiliations:** 1 Institute of Bioinformatics, International Technology Park, Bangalore, Karnataka, India

Accepted.

Are the conclusions about the tool and its performance adequately supported by the findings presented in the article?

Yes

Is the rationale for developing the new software tool clearly explained?

Yes

Is the description of the software tool technically sound?

Yes

Are sufficient details of the code, methods and analysis (if applicable) provided to allow replication of the software development and its use by others?

Yes

Is sufficient information provided to allow interpretation of the expected output datasets and any results generated using the tool?

Yes

Reviewer Expertise:

Multiomics, genomics and ML

I confirm that I have read this submission and believe that I have an appropriate level of expertise to confirm that it is of an acceptable scientific standard.

# Peer review: Reviewer response for version 2

**DOI:** 10.5256/f1000research.31433.r76607

**Published:** 2021-01-11

**Authors:** Osman Ugur Sezerman

**Affiliations:** 1 Department of Biostatistics and Bioinformatics, Life Science Institute, Acıbadem University, Istanbul, Turkey

I am satisfied with the changes.

Are the conclusions about the tool and its performance adequately supported by the findings presented in the article?

Yes

Is the rationale for developing the new software tool clearly explained?

Yes

Is the description of the software tool technically sound?

Yes

Are sufficient details of the code, methods and analysis (if applicable) provided to allow replication of the software development and its use by others?

Partly

Is sufficient information provided to allow interpretation of the expected output datasets and any results generated using the tool?

Yes

Reviewer Expertise:

Bioinformatics

I confirm that I have read this submission and believe that I have an appropriate level of expertise to confirm that it is of an acceptable scientific standard.

# Peer review: Reviewer response for version 1

**DOI:** 10.5256/f1000research.29685.r73698

**Published:** 2020-12-14

**Authors:** Abhishek Kumar

**Affiliations:** 1 Institute of Bioinformatics, International Technology Park, Bangalore, Karnataka, India

Yousef *et al.* have presented SVM-RCE-R as the improved implementation of the original algorithm SVM-RCE in Knime with additional features. The most important new feature is the ranking function R(). The authors demonstrate R() is important in improving the performance over the original study SVM-RCE. The Knime implementation demonstrates the novelty of the integration of biological information into the machine learning feature selection component. The approach is performing grouping by clustering and ranking by internal cross-validation. The approach is interesting and would also contribute to the new trend of integrative biological knowledge into the process of feature sections.

Major issues:

 I have two major concerns:

1. The authors must define the "user-specific ranking function" more clearly at the start of the manuscript.
2. Usage of collected datasets are not clear and authors must provide better examples of results derived from datasets.

Minor issues:

 The authors must improve carefully figure and table legends plus typos and errors:

1. Table 3 title: 'ACC=accuracy', 'Spe=specificity', and so on.
2. Figure 4: "The average of 100 iterations if computed” should be is 'computed'.
3. Results section: "For the comparison of the three approaches, five datasets are considered for," -> the last 'for' should be removed.
4. Figure 3: It should be 'SVM-RCE-R' and not "SVM-RCE".
5. Figure 1: It should be 'SVM-RCE-R' and not "SVM-RCE".

Are the conclusions about the tool and its performance adequately supported by the findings presented in the article?

Yes

Is the rationale for developing the new software tool clearly explained?

Yes

Is the description of the software tool technically sound?

Yes

Are sufficient details of the code, methods and analysis (if applicable) provided to allow replication of the software development and its use by others?

Yes

Is sufficient information provided to allow interpretation of the expected output datasets and any results generated using the tool?

Yes

Reviewer Expertise:

Multiomics, genomics and ML

I confirm that I have read this submission and believe that I have an appropriate level of expertise to confirm that it is of an acceptable scientific standard, however I have significant reservations, as outlined above.

# Response

**Published:** 2020-12-15

**Authors:** Malik Yousef

**Affiliations:** Zefat Academic College, Israel

Dear Dr. Abhishek Kumar,

                 Thank you very much for your feedback suggestions, which significantly improve the manuscript and enrich the content. We would like to address your comments:

- *The authors must define the "user-specific ranking function" more clearly at the start of the manuscript.*

We have now made changes based on the earlier reviewers as well to clarify and focus the manuscript more on the user-specific ranking function. We have made the relevant changes in the introduction, abstract as well as defining a more focused heading on the novel ranking feature.

- *Usage of collected datasets are not clear and authors must provide better examples of results derived from datasets.*

We noticed that we were not clear in describing which datasets were used for which figures, therefore now we have mentioned in detail which datasets are used in their relevant tables and figures.

- *Figure 4: "The average of 100 iterations if computed” should be is 'computed'.*

We wrote it down as “if” since the user has the option of computing the algorithm to his/her needs and it can differ to their choices.

 We have also gone through all the typos and spelling mistakes that you have so kindly provided us, thank you very much. Please let us know if you further feedback.

 Yours truly,

 The authors.

# Peer review: Reviewer response for version 1

**DOI:** 10.5256/f1000research.29685.r73308

**Published:** 2020-12-02

**Authors:** Osman Ugur Sezerman

**Affiliations:** 1 Department of Biostatistics and Bioinformatics, Life Science Institute, Acıbadem University, Istanbul, Turkey

In this manuscript, the authors describe the implementation of feature selection method, SVM-RCE-R, in KNIME and demonstrate the usefulness of this tool. As they clearly describe, this is an improvement of the previously developed SVM-RCE. The most important novel feature in SVM-RCE-R is the user-specific ranking function, allowing the researcher to select for different performance metrics. As KNIME provides an easy-to-use interface, and the tool requires simple input formats, it will most likely be a valuable tool for biomedical researchers with many different backgrounds.

**Major issues:**

- The introduction of the user-specific ranking function could be emphasized more clearly, perhaps in the Introduction section.
- The analyzed datasets presented in the results section should be more clearly defined for each result, the number of datasets are confusing.
- The abstract states that “The input for the Knime tool is an EXCEL file (or text or CSV) with a simple structure…”, this structure should be described in the main text.

**Minor issues:**

- In Table 3, the metric names should be replaced by the weights of these metrics.
- Minor typos:
   
  - Page 3, last paragraph: it should be “user-specific”, “ranking functions” should be singular (i.e., “ranking function”).
  - Page 4, in the user-specific ranking function, “pres” should be “prec”.
  - Page 5, “Reserve” should be “Rserve”.
  - Please use "meta-node" throughout the manuscript.
  - “are under curve” should be “area under the curve”.

Are the conclusions about the tool and its performance adequately supported by the findings presented in the article?

Yes

Is the rationale for developing the new software tool clearly explained?

Yes

Is the description of the software tool technically sound?

Yes

Are sufficient details of the code, methods and analysis (if applicable) provided to allow replication of the software development and its use by others?

Partly

Is sufficient information provided to allow interpretation of the expected output datasets and any results generated using the tool?

Yes

Reviewer Expertise:

Bioinformatics

I confirm that I have read this submission and believe that I have an appropriate level of expertise to confirm that it is of an acceptable scientific standard, however I have significant reservations, as outlined above.

# Response

**Published:** 2020-12-13

**Authors:** Malik Yousef

**Affiliations:** Zefat Academic College, Israel

Dear Prof. Ugur Sezerman,

 We deeply appreciate your taking the time to provide your valuable feedback to us. We have revised the manuscript based on that feedback and would also like to address the comments:

- *The introduction of the user-specific ranking function could be emphasized more clearly, perhaps in the Introduction section.*

We have now included a description of the novelty of our ranking function in the abstract to better focus on this topic. Moreover, we have also updated the introduction section with your recommendations. Finally, we have revised the methods section so that it clearly states and describes the user specific ranking function.

- *The analyzed datasets presented in the results section should be more clearly defined for each result, the number of datasets are confusing.*

We have updated the results section and now clearly mention which datasets were used in each graph or table. We have also included how many datasets were used for comparison results and have clearly stated their names.

- *The abstract states that “The input for the Knime tool is an EXCEL file (or text or CSV) with a simple structure…”, this structure should be described in the main text.*

In the data section, we now include a description of the input data, and we have also updated the underlying data for the input data files.

 As for the minor comments, all the relevant mistakes pointed out have been corrected and updated. We once again thank you for your feedback and contribution and we welcome any further feedback.

 Kind regards,

 The authors

# Peer review: Reviewer response for version 1

**DOI:** 10.5256/f1000research.29685.r73564

**Published:** 2020-12-02

**Authors:** Marco Chierici

**Affiliations:** 1 Fondazione Bruno Kessler, Trento, Italy

The authors describe the Knime implementation of SVM-RCE-R, an algorithm for feature ranking and selection that is described as an improved version of the previously developed SVM-RCE. The motivation behind this new implementation is clearly stated and mainly resides in Knime’s user-friendliness and ease of use, besides Knime being an Open Source project. Because of this, the software has the potential to be a valuable tool for researchers in the computational biology community.

 My first concern is about potential confusion: it is not clear whether the manuscript focuses on the Knime implementation of a previously devised algorithm (SVM-RCE-R) or whether it introduces both the algorithm and its implementation. I think this aspect should be better stated in both the Abstract and the Introduction.

 My other concerns follow.

**Conveyance of novelty:**

- The novelty of SVM-RCE-R vs. SVM-RCE, which, to my understanding, is the introduction of the ranking functions, should be pointed out more effectively. In particular, the Methods section opens with the description of the SVM-RCE algorithm: this comes unexpectedly to the reader since the manuscript should focus on describing SVM-RCE-R. The novelty element sneaks into the “Weighted rank function” subsection, while it should be better highlighted. This could be achieved by merely restructuring the Methods section or by adjusting the subsection titles.

**Data and analysis details:**

- It has to be clearly stated which datasets are used for each experiment discussed in the Results section: as far as I have understood, the experiments concerning the ranking functions (e.g., Figure 4) consider all 12 datasets, while those comparing the three approaches (e.g., Table 3) consider five datasets. Still, Figure 5 presents results for three datasets while the main text states “for each data set”. The beginning sentence of Results can be misleading, as it may seem that all results are obtained on five datasets.
- Were the GEO datasets used “as is” or was some preprocessing applied? More details should be included regarding the input format: for example, is it a gene expression table? What should be on the rows and the columns? Should there be row and column names?

**Presentation of results:**

- A comparison with SVM-RCE is only briefly mentioned, while the discussion would benefit from a more extended comparison (as done with SVM-RFE and SVM-IG), also to show SVM-RCE-R improvements over the previous version.
- The authors state that “SVM-RFE has a slightly better accuracy although significantly lower specificity than SVM-RCE-R”, but according to Table 3 it appears that average SVM-RFE accuracy is always lower than, or equal to, average SVM-RCE-R accuracy, and the same for SVM-RFE specificity (except for one dataset): please elaborate. Moreover, how was the significance assessed?
- For the ranking function, the authors chose accuracy, sensitivity, specificity, F1 score, AUC, and precision. This is quite a comprehensive set of metrics, which the authors could further enrich with the Matthews Correlation Coefficient (MCC), a balanced measure of accuracy and precision that can still be effectively used when sample classes are highly imbalanced.
- The notation used in Table 3 should be improved: it is misleading to indicate the weights using the metric names to which they refer. For example, Acc=0.2 should be replaced by w _1_=0.2, and so on. Please refer to the rank function notation introduced in “Weighted rank function” and rename the weights accordingly.
- Figure 4: since the goal is to compare different ranking functions, and the ranking function includes F1 among its terms, I suggest adding the average F1 to the figure. The same holds for Figure 5.

**Misc and minors:**

 In general, please consider having the paper proof checked by a native English speaker to improve overall readability and address typos.

- Please consider improving the quality of Figures, especially 1-3.
- Please indicate the measure of dispersion represented by the error bars in the Figures.
- I am afraid the following sentence is not correct: “As illustrated in Pseudocode 1, in the original code of SVM-RCE we used the accuracy as the determinant for ranking the clusters.” There is no mention of accuracy in Pseudocode 1, only “performance”. Moreover, in Pseudocode 1 please consider improving “t = Test classifier on Xv –calculate performance” as it is not clear, and please change “aggregation the scores” to “aggregating the scores”.
- In Methods (p. 3): “test set performance” would be more accurate than “training-testing performance”.
- In Methods (p. 4): probably “prec” should be used instead of “pres”.
- In “Implementation in Knime”: the sentence “by configuring the node Counting Loop Start” more likely belongs to the end of the preceding paragraph.
- Figure 1 caption: please change “RCE based SVM” to “SVM-RCE” for consistency; please address the typo “compenents”.
- In “Operation” (p. 5): “Reserve” should be changed to “Rserve”.
- In Results (p. 5): please check “five datasets are considered for”.
- In Results (p. 6): “... six rank functions as listed in Table 2”; it should be Table 3.
- In Results (p. 9): please check “with 0.6 and weight 0.4”.
- Please use either “meta node” or “meta-node” throughout the manuscript; the same holds for “SVM IG” vs SVM-IG.
- Please use “area under the curve” instead of “area under curve” (also in Figures).
- Please change w_1, w_2, … and w1,..,w6 to w _1_, w _2_, … (Results); please mind proper spacing and ellipsis in “w1,..w6”.

Are the conclusions about the tool and its performance adequately supported by the findings presented in the article?

Yes

Is the rationale for developing the new software tool clearly explained?

Yes

Is the description of the software tool technically sound?

Yes

Are sufficient details of the code, methods and analysis (if applicable) provided to allow replication of the software development and its use by others?

Yes

Is sufficient information provided to allow interpretation of the expected output datasets and any results generated using the tool?

Partly

Reviewer Expertise:

Bioinformatics, artificial intelligence, computational biology

I confirm that I have read this submission and believe that I have an appropriate level of expertise to confirm that it is of an acceptable scientific standard, however I have significant reservations, as outlined above.

# Response

**Published:** 2020-12-13

**Authors:** Malik Yousef

**Affiliations:** Zefat Academic College, Israel

Dear Marco Chierici,

 Thank you for your constructive feedback. We found your insights helpful for improving the clarity of the manuscript. Below, we have addressed each of the items raised:

- *My first concern is about potential confusion: it is not clear whether the manuscript focuses on the Knime implementation of a previously devised algorithm (SVM-RCE-R) or whether it introduces both the algorithm and its implementation. I think this aspect should be better stated in both the Abstract and the Introduction.*

We have updated the abstract as well as the introduction to describe the novelty of the user specific ranking function as well the simplicity of KNIME implementation.

- *The novelty of SVM-RCE-R vs. SVM-RCE, which, to my understanding, is the introduction of the ranking functions, should be pointed out more effectively. In particular, the Methods section opens with the description of the SVM-RCE algorithm: this comes unexpectedly to the reader since the manuscript should focus on describing SVM-RCE-R. The novelty element sneaks into the “Weighted rank function” subsection, while it should be better highlighted. This could be achieved by merely restructuring the Methods section or by adjusting the subsection titles.*

In response to this suggestion, we have now changed the headings in the methods section. We now we have a specific subsection to bring the reader’s attention to the user specific ranking function.

- *It has to be clearly stated which datasets are used for each experiment discussed in the Results section: as far as I have understood, the experiments concerning the ranking functions (e.g., Figure 4) consider all 12 datasets, while those comparing the three approaches (e.g., Table 3) consider five datasets. Still, Figure 5 presents results for three datasets while the main text states “for each data set”. The beginning sentence of Results can be misleading, as it may seem that all results are obtained on five datasets.*

We have now made it clear in the results section about which datasets were used for which figures. Moreover, it is clearly stated results that we have used all the datasets for our results and specific datasets were used for comparison.

  

- *Were the GEO datasets used “as is” or was some preprocessing applied? More details should be included regarding the input format: for example, is it a gene expression table? What should be on the rows and the columns? Should there be row and column names?*

Based on your comment we have now included a description of the dataset as well. In addition, we have included the input data in our underlying data so that the results can easily be replicated.

- *A comparison with SVM-RCE is only briefly mentioned, while the discussion would benefit from a more extended comparison (as done with SVM-RFE and SVM-IG), also to show SVM-RCE-R improvements over the previous version.*

We agree, the section comparing SVM-RCE and SVM-RCE-R is not clearly mentioned. We have now included a sub section to indicate to the readers the comparison results.

- *The authors state that “SVM-RFE has a slightly better accuracy although significantly lower specificity than SVM-RCE-R”, but according to Table 3 it appears that average SVM-RFE accuracy is always lower than, or equal to, average SVM-RCE-R accuracy, and the same for SVM-RFE specificity (except for one dataset): please elaborate. Moreover, how was the significance assessed?*

We have corrected the mistake which in the results for Table 3 and we pointed out that only SVM-RCE-R performs weaker in sensitivity for two of the datasets otherwise it outperforms or is on par with other approaches.

  

- *For the ranking function, the authors chose accuracy, sensitivity, specificity, F1 score, AUC, and precision. This is quite a comprehensive set of metrics, which the authors could further enrich with the Matthews Correlation Coefficient (MCC), a balanced measure of accuracy and precision that can still be effectively used when sample classes are highly imbalanced.*

We are planning to use the MCC in our further research where we search for the optimal combination of ranks. As you have mentioned it is a very good metric for highly imbalanced datasets. Thank you very much for the feedback about the metric.

  

- *The notation used in Table 3 should be improved: it is misleading to indicate the weights using the metric names to which they refer. For example, Acc=0.2 should be replaced by w1=0.2, and so on. Please refer to the rank function notation introduced in “Weighted rank function” and rename the weights accordingly.*

We have now updated the results in the table so that it is consistent with what is stated in the description of the ranking function.

  

- *Figure 4: since the goal is to compare different ranking functions, and the ranking function includes F1 among its terms, I suggest adding the average F1 to the figure. The same holds for Figure 5.*

We were focusing on the more widely used metrics to show the performance of our models, but as you have mentioned also looked into F-measure, so we now have included it in our results. All other typos and errors that you have so helpfully provided have been corrected in the revised manuscript.

 Kind regards,

 The authors
